# 

**Published:** 1896-08

**Authors:** 


### TABLE OF CONTENTS.

ORIGINAL COMMUNICATIONS. PAGE

Clinical Considerations on Infectious Gingivites. By CHARLES GREENE CUMSTON, B.M.S., M.D. . . . . . . . . . . . . . . . . . . . . . . . 481

A System of Detachable Facings. By W. L. MASON, D.D.S., Red Bank, N. J. . . . . . . . . . . . . . . . . . . . . . . . . . . . . . . 488

The Future of Dentistry electrically considered. By G. H. GUY, New York 491

An Oral Camera for Röntgen Photography. By WILLIAM ROLLINS, Boston, Mass. . . . . . . . . . . . . . . . . . . . . . . . . . . . . . 497

The Qualifications Necessary for the Dental Practitioner. By DR. P. T. SMITH, Denver, Col. . . . . . . . . . . . . . . . . . . . . . . . . 500

ABSTRACTS AND TRANSLATIONS.

A Case of Calcification of a Widely Exposed Pulp. By MR. CHARLES S. TOMES . . . . . . . . . . . . . . . . . . . . . . . . . . . . . 503

REPORTS OF SOCIETY MEETINGS.

American Academy of Dental Science . . . . . . . . . . . . . . . . . . 508

Academy of Stomatology . . . . . . . . . . . . . . . . . . . . . . . . 517

Central Dental Association of Northern New Jersey . . . . . . . . . . . 527

EDITORIAL.

An Old Subject revived . . . . . . . . . . . . . . . . . . . . . . . . 542

Dr. Williams and the Association of Faculties . . . . . . . . . . . . . 545

OBITUARY.

Dr. C. W. Spalding . . . . . . . . . . . . . . . . . . . . . . . . . . 547

CURRENT NEWS.

Harvard Dental Alumni Association . . . . . . . . . . . . . . . . . . 548

